# Impact of dose allocation between high‐ and low‐kV scans on virtual monoenergetic image quality in developmental dual‐energy cone‐beam CT for image‐guided radiotherapy

**DOI:** 10.1002/acm2.70545

**Published:** 2026-04-27

**Authors:** Andrew Keeler, Luke Layman, Ha Nguyen, Jason Luce, Mathias Lehmann, John C. Roeske, Hyejoo Kang

**Affiliations:** ^1^ Department of Radiation Oncology, Stritch School of Medicine, Cardinal Bernardin Cancer Center Loyola University of Chicago Maywood Illinois USA; ^2^ Varian Imaging Laboratory Baden Switzerland

**Keywords:** cone‐beam CT, dual‐energy, virtual monoenergetic

## Abstract

**Background:**

Dual‐energy cone‐beam CT (DE‐CBCT) offers potential as an advanced imaging technique for image‐guided radiation therapy (IGRT) through the production of virtual monoenergetic images (VMI). CBCT‐VMI can show enhanced image quality, with improved CNR and reduced nonuniformity artifacts, compared to polychromatic images. However, optimized DE‐CBCT protocols are required for clinical application. In particular, the impact of the dose allocation between the two acquisition energies on the resulting VMI image quality remains to be assessed.

**Purpose:**

This study evaluates the impact of different dose allocation strategies between the low‐ and high‐energy scans on the image quality of DE‐CBCT derived VMI.

**Methods:**

A series of seven DE‐CBCT scans were performed using various dose allocations ranging from 10% Low‐energy/90% High‐energy (LE/HE) to 70%/30% LE/HE in 10% intervals while maintaining a constant total imaging dose. To assess the impact of the dose allocation strategies, VMI were reconstructed at 40, 60, 80, and 100 keV and evaluated for Hounsfield Unit (HU) accuracy, relative contrast‐to‐noise ratio (rCNR) enhancement, and HU uniformity compared to a dose‐matched clinical CBCT protocol.

**Results:**

DE‐CBCT protocols between 20%/80% and 70%/30% LE/HE showed significant improvements over the clinical protocol, with CNR values improving by an average of 18%–32% at 60 keV and non‐uniformity artifacts reduced to negligible levels without loss of HU accuracy. Statistical comparison between these DE‐CBCT protocols further showed that the specific dose allocation had statistically insignificant impact on the resulting VMI image quality between 20%/80% and 50%/50% LE/HE at any energy. Of the remaining DE protocols, 70%/30% showed a significant decrease in HU accuracy while 10%/90%, 60%/40%, and 70%/30% showed reduced rCNR enhancement at some energies.

**Conclusions:**

Overall, a wide range of dose allocations are shown to produce comparably near‐optimal VMI quality. This finding supports protocol design flexibility for future clinical DE‐CBCT implementations.

## 1 INTRODUCTION

Dual‐energy cone‐beam computed tomography (DE‐CBCT) is an emerging imaging technique with the potential to enhance image‐guided radiotherapy (IGRT) by enabling the reconstruction of virtual monoenergetic images (VMIs).^1^, ^2^ VMIs have been shown to offer enhanced soft‐tissue contrast, improved Hounsfield Unit (HU) accuracy, and reduced beam‐hardening artifacts compared to polychromatic CBCT,^1^, ^3^, ^4^, ^5^, ^6^ making DE‐CBCT a promising advancement in image‐guided workflows. While clinical implementation of DE‐CBCT remains in development, active research is ongoing to refine acquisition,^4^, ^7^, ^8^, ^9^, ^10^ decomposition,^1^, ^8^, ^10^, ^11^, ^12^, ^13^ and reconstruction^4^, ^14^ techniques to facilitate smooth integration into clinical practice.

Questions about how to most effectively allocate the combined imaging dose between the low‐energy (LE) and high‐energy (HE) scans naturally arise during the development of DE‐CBCT protocols. While several past studies have assessed the impacts of dose allocation on various DE‐CT and DE‐CBCT image types,^1^, ^6^, ^15^, ^16^, ^17^, ^18^, ^19^ the dose allocation guidance offered by these past works is inconsistent and task‐specific.^17^ To date, no focused studies have assessed the impact of dose allocation on CBCT‐based VMI. This lack of clear guidance regarding optimal allocation strategies for CBCT‐VMI would present difficulties as DE‐CBCT continues to be developed toward clinical applications.

To address this issue, this study aims to evaluate the impact of dose allocation on VMI image quality in DE‐CBCT. Our findings will help guide DE‐CBCT protocol design and support the future translation of this technology into clinical IGRT workflows.

## 2 METHODS AND MATERIALS

### 2.1 Data acquisition

CBCT images of a Catphan 604 phantom (The Phantom Laboratory, Salem, NY) were obtained using the onboard imager (OBI) of a Varian TrueBeam linear accelerator system (Varian Medical Systems, Palo Alto, CA). DE data was acquired using sequential scans of LE (80 kV) and HE (140 kV) settings in treatment mode. Scans were performed using half‐fan geometry at the maximum frame rate of 15 fps. Due to current‐time product (mAs) limits on the TrueBeam system (see Section 2.2), the cumulative dose for each protocol was set to approximately 1/3 that of the standard 140 kV Pelvis‐Large protocol (Table 1). A clinical CBCT scan protocol (CSP) was also acquired at 140 kV on the same system with the same cumulative imaging dose to serve as a reference image.

**TABLE 1:** Combinations of kV, mA, and ms settings used for the CSP and each DE protocol. The 70% LE scan represented the maximum mAs allowed by the TrueBeam system, which limited the imaging dose employed in this study.

Protocol (LE/HE)	80 kV			140 kV			CBDI Sum (mGy)
	mA	ms	Est. CBDI (mGy)	mA	ms	Est. CBDI (mGy)	
CSP (−/100%)	–	–	–	24	25	23.0	23.0
10%/90%	36	25	2.2	22	25	20.8	23.0
20%/80%	72	25	4.3	19	25	18.1	22.4
30%/70%	115	25	6.9	16	25	15.7	22.6
40%/60%	153	25	9.2	14	25	13.8	23.0
50%/50%	160	30	11.6	14	21	11.8	23.4
60%/40%	160	36	13.9	10	21	8.7	22.6
70%/30%	160	42	16.2	16	10	6.9	23.1

### 2.2 Dose allocation strategies

In principle, creating a DE image with a large proportion of the total dose in the LE scan would produce an image with improved contrast driven by a high proportion of photoelectric interactions, but with sharply increased noise. Conversely, allocating a larger portion of dose to the HE scan would increase the proportion of Compton interactions, reducing image noise, but with reduced overall contrast.^1^ We seek to determine optimal combinations of LE and HE dose for contrast‐to‐noise ratio (CNR) enhancement and beam hardening reduction.

Seven dose allocations between the LE and HE scans were evaluated to empirically locate optimal dose allocation values: 10%/90%, 20%/80%, 30%/70%, 40%/60%, 50%/50%, 60%/40%, and 70%/30% (LE/HE). Since the TrueBeam mAs was limited to between 0.1 and 6.7 mAs per projection, dose allocations of more than 70% LE were unable to be acquired. Relative imaging doses for each acquisition were estimated by defining linear correlations between the scan mAs at each kV energy and the corresponding cone‐beam dose index (CBDI) measured in a 160 mm head phantom, as described by Gros et al.^20^

### 2.3 Image reconstruction and VMI generation

Preprocessing, including beam hardening correction for the CSP, projection‐domain material decomposition, image reconstruction, and HU mapping of the CSP were performed using the Matlab‐based TIGRE (Tomographic Iterative GPU‐based Reconstruction) toolkit.^3^, ^21^, ^22^ VMIs with various energies from 40–100 keV were generated using the Dual‐energy Image Synthesizer for CBCT (DISC)^5^ and mapped to HU using an energy‐dependent calibration function.

### 2.4 Image quality evaluation and statistical analysis

Image quality of the CSP and DE protocols was assessed using HU accuracy, CNR enhancement, and HU uniformity. HU accuracy and CNR enhancement were assessed at 40, 60, 80, and 100 keV, with *t*‐tests assessing statistical improvements of DE protocols over the CSP, and Analysis of Variance (ANVOA) measuring variability between DE protocols. All statistical analysis was conducted using a 5% significance level.

**FIGURE 1:**
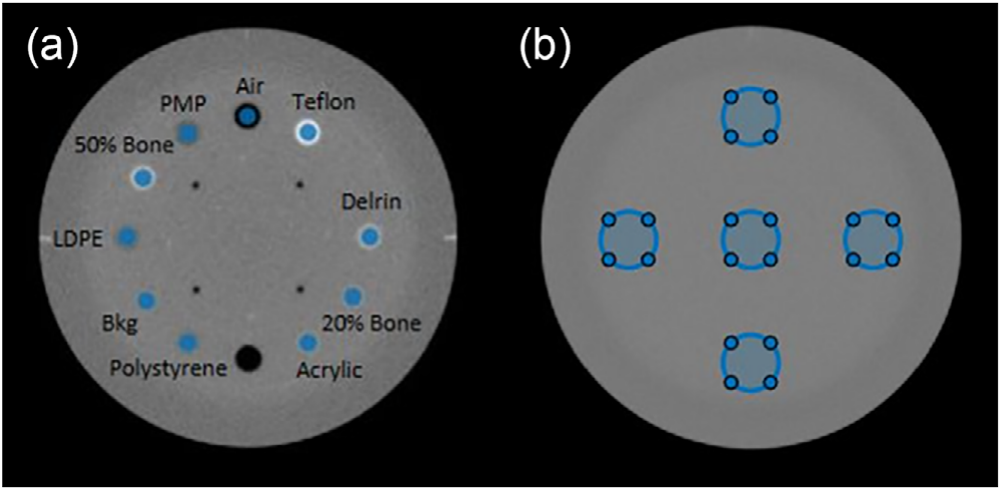
ROIs used in analysis of image quality. (a) Material inserts representing a range of HU values. Used for assessing HU accuracy and rCNR enhancement. (b) Uniform material used to assess HU uniformity. ROI: Region of Interest, HU: Hounsfield Unit, rCNR: relative contrast‐to‐noise ratio.

- **HU accuracy**: Mean Hounsfield Unit (HU) values were measured for each material insert using circular regions of interest (ROIs) and compared to theoretical HU values for the material inserts (Figure 1a). The theoretical HU values for the VMI are computed energy‐by‐energy from the material‐specific attenuation values provided by the phantom manufacturer. Mean absolute HU errors (MAE) and standard deviations were compared to assess statistical variation between protocols.
- **CNR enhancement**: For each material insert, CNR was calculated as
(1) $$\;\mathrm{CN}R_{i}=\frac{\left|HU_{i}-HU_{\mathrm{bkg}}\right|}{\sqrt{\frac{1}{2}\left(\sigma_{i}^{2}+\sigma_{\mathrm{bkg}}^{2}\right)}}$$

with ***i*** representing the material ROI, ***bkg*** representing the background material ROI, ***HU*** representing the mean HU value, and ***σ*** representing the standard deviation of the HU value over the ROI.^5^ The relative CNR enhancement (rCNR) of DE protocol ***P*** over the CSP was defined as

(2) $$\;rCNR_{i,P}=\frac{CNR_{i,P}}{CNR_{i,CSP}}\;$$

- **HU uniformity**: VMI created with projection‐domain methods are theoretically expected to eliminate non‐uniformity artifacts attributable to beam hardening due to the monoenergetic nature of the virtual X‐ray source.^1^, ^5^ In practice, uncertainties in the VMI synthesis can result in some beam hardening artifacts nevertheless appearing, but appropriate energy selection can substantially reduce or eliminate them.^1^ Here, HU uniformity for all protocols was assessed at 65 keV, identified as the optimal energy for image uniformity across all protocols using the inbuilt DISC functions.^5^

Five circular ROIs were placed in the uniformity module of the phantom, located at the center, top, bottom, left, and right of the phantom, as shown in Figure 1b. Image uniformity was assessed as the maximum difference in HU between the peripheral ROIs and the central ROI. These values are compared to the image noise, with a maximum deviation larger than the noise level indicating the presence of nonuniformity artifacts.

## 3 RESULTS

**FIGURE 2:**
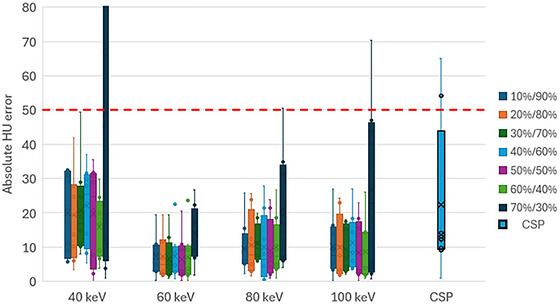
HU errors for the material inserts for the CSP and DE protocols. The red dashed line indicates the TrueBeam OBI HU accuracy tolerance.

As illustrated in Figure 2, all DE protocols except 70%/30% improved HU accuracy compared to the CSP at all energies. MAEs at each energy were statistically similar for all DE protocols except 70%/30% (*p* = 0.95, 1.00, 0.99, and 0.99, respectively). 70%/30% HU accuracy ranged from visibly but insignificantly worse than other DE protocols (60 keV) to exceeding the 50 HU tolerance for the TrueBeam OBI (40 keV).

**FIGURE 3:**
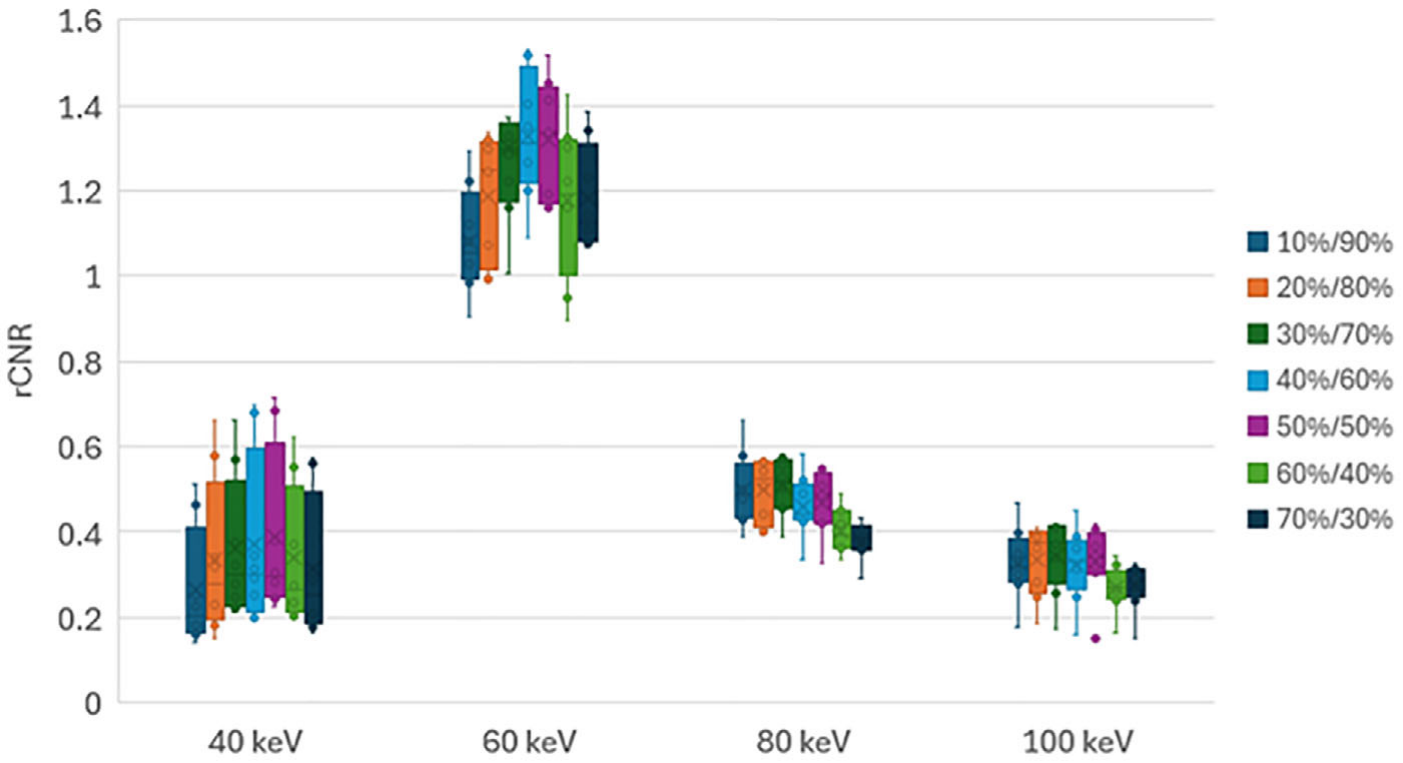
rCNR enhancement for each DE protocol.

All DE protocols show mean CNR enhancement over the CSP at 60 keV, with DE protocols from 20%/80% to 70%/30% showing significant enhancement (*p* < 0.005 for each). In terms of relative DE protocol performance, significant dips in rCNR are present for the 10%/90% protocol at 60 keV and 60%/40% and 70%/30% protocols at 80 keV (*p* = 0.02, 0.05, and 0.02). Visually similar though insignificant rCNR reductions are present for those protocols at other energies (Figure 3). The range of 20%/80% to 50%/50% protocols provide consistently optimal CNR enhancement across all energies. (*p* = 0.96, 0.30, 0.53, and 0.96 respectively)

**FIGURE 4:**
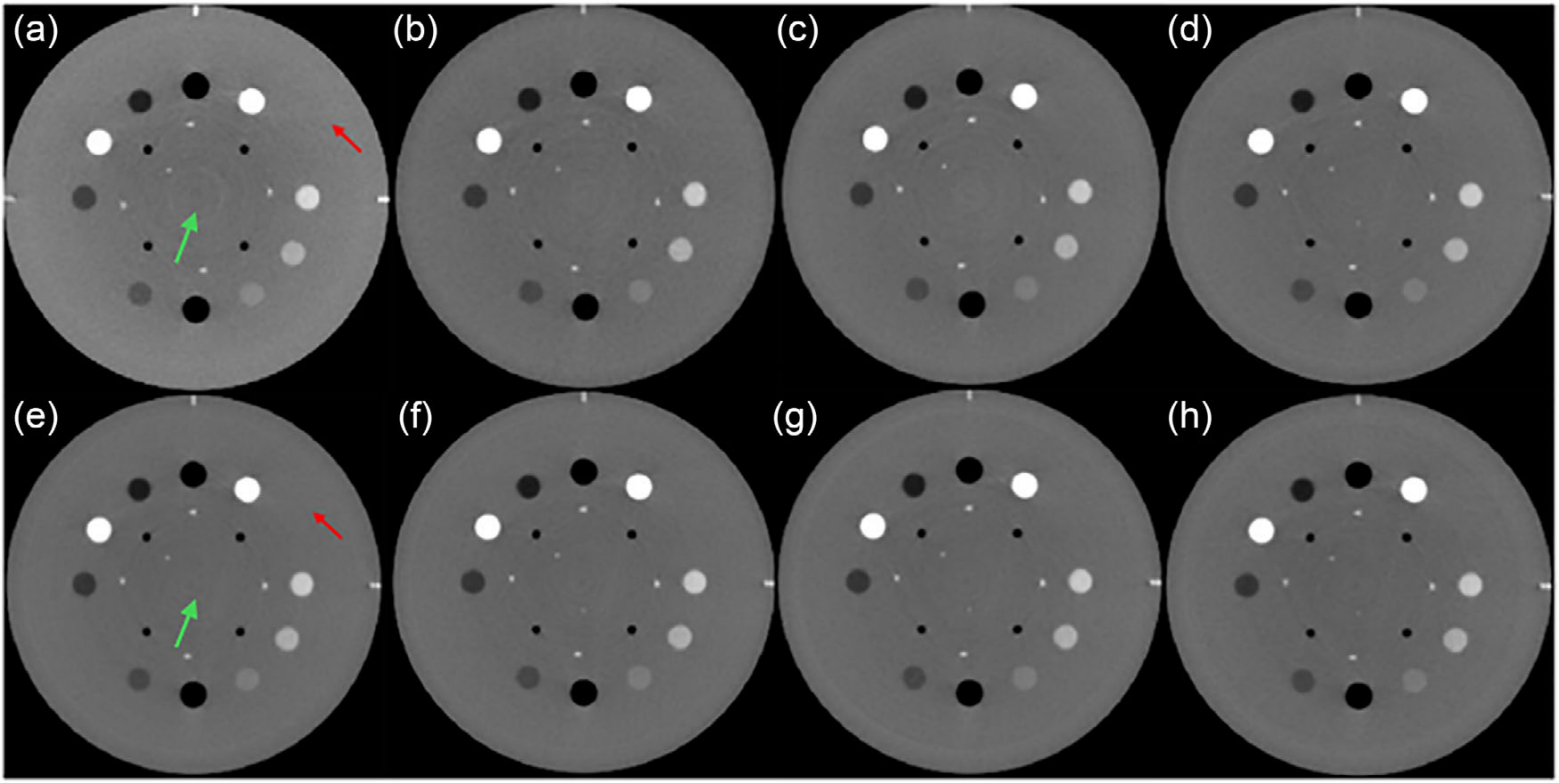
Material insert module of the Catphan 604, imaged using the (a) CSP, (b) 10%/90%, (c) 20%/80%, (d) 30%/70%, (e) 40%/60%, (f) 50%/50%, (g) 60%/40%, and (h) 70%/30% protocols at 65 keV. The CSP shows lighter shading around the periphery of the phantom body (**red arrow**) compared to the center (**green arrow**), indicating a cupping artifact due to beam hardening which is substantially absent from the DE protocols. Window/Level: 800/100

All DE protocols either sharply reduce (70%/30%) or remove the cupping artifact observed in the CSP scan (Figure 4). The maximum HU deviation observed in the uniformity test for the CSP was 21.4 HU, much larger than the 9 HU noise level. By comparison, the 70%/30% protocol showed a maximum deviation of 9.8 HU and the remaining DE protocols showed negligible nonuniformity compared with the approximately 7 HU noise levels observed in the VMI. (Table 2)

**TABLE 2:** HU uniformity measure for the CSP and DE protocols.

Protocol (LE/HE)	HU nonuniformity (HU)	Noise level (HU)
CSP	21.4	9.2
10%/90%	3.7	8.0
20%/80%	5.5	7.3
30%/70%	3.1	7.2
40%/60%	2.0	6.7
50%/50%	3.4	6.3
60%/40%	3.8	6.1
70%30%	8.6	6.3

## 4 DISCUSSION

Dual‐energy imaging raises important questions about how to most effectively allocate imaging dose between the dual‐energy scans to optimize image quality. However, past studies in DE‐CBCT have provided inconsistent guidance about optimal strategies for allocating dose.^1^, ^6^, ^15^, ^16^, ^17^, ^18^, ^19^ For example, Gang et al.^6^ recommended dose partitions that favored additional dose in the high‐energy scan to maximize detectability for certain tasks, while Li et al.^1^ and Li et al.^15^ found that allocating additional dose to the low‐energy scan provided improved CNR. Similarly, studies that look at dose allocation in DE‐CT recommend strategies that can be inconsistent and task‐specific.^17^, ^18^ However, consideration of VMI is absent from most of these discussions surrounding optimal dose allocation in DE‐CBCT and DE‐CT.

This study seeks to fill this gap by directly assessing the effects of dose allocation on VMI image quality in a clinical setting for DE‐CBCT. We show that allocations from 20%/80% to 50%/50% show significantly improved CNR values at 60 keV compared with the dose‐matched CSP. Additionally, allocations from 10%/90% to 60%/40% show comparable HU accuracy to the CSP regardless of energy and all DE protocols show improved uniformity. Overall, these findings suggest that a wide range of dose allocations, from at least 20%/80% to 50%/50%, produce VMI with similar image quality improvements over the comparably‐dosed CSP. A wider range of allocations, potentially encompassing 10%/90% and 60%/40% protocols, shows similar improvements for at least some VMI energies. Due to the prior lack of guidance for allocating dose for CBCT‐VMI, these results should serve as a first step in designing appropriate scanning protocols for clinical DE‐CBCT imaging.

These findings highlight both an important finding and a major limitation of this study. The finding of a broad range of near‐optimal dose allocations supports flexibility in designing DE‐CBCT protocols, as physical limitations such as the mAs limits encountered in this work or the need for motion management during acquisition can be accommodated without compromising VMI image quality. However, these findings may be limited by a lack of generalizability. VMI image quality is affected by the specific dual X‐ray spectra used to generate the images, meaning that changing the acquisition method or the OBI may impact the resulting image quality. While techniques such as kV switching^4^ or dual‐source acquisitions^1^ may be sufficiently similar to the sequential scan methods used here to warrant similar allocations, techniques such as beam modulation,^7^, ^8^ multi‐layer detectors,^9^, ^10^ or even varying the kV settings for the acquisition or using OBI from different manufacturers may produce different allocation preferences than observed in this work.

Another limitation of this study is the reliance on a small, geometrical phantom. While the Catphan 604 has the advantage of having well‐characterized sensitometry and uniformity modules for quantitative analysis, the small size and limited anatomical realism of the phantom provides limited insight into how DE‐CBCT would impact the treatment of patients clinically. Future work will involve testing with larger, more realistic phantoms to develop specific DE‐CBCT imaging protocols. This can be followed by patient studies to directly assess the impacts of CBCT‐VMI on clinical decision‐making in IGRT.

## 5 CONCLUSIONS

This work investigates the effect of dose allocation on the image quality of VMI derived from DE‐CBCT. We find that a wide range of allocation strategies between at least 20%/80% and 50%/50% LE/HE provide near‐optimal image enhancement over clinical protocols, with a wider range of protocols potentially showing similar enhancements at certain VMI energies. These findings support flexibility in protocol development as DE‐CBCT moves towards clinical implementation and application in IGRT.

## AUTHOR CONTRIBUTIONS

**Andrew Keeler**: Research conceptualization, Methodology contribution, Manuscript preparation. **Luke Layman**: Methodology contribution. **Ha Nguyen**: Methodology contribution, Manuscript preparation. **Jason Luce**: Methodology contribution, Manuscript preparation. **Mathias Lehmann**: Methodology contribution, Manuscript preparation. **John C. Roeske**: Methodology contribution, Manuscript preparation. **Hyejoo Kang**: Methodology contribution, Manuscript preparation

## CONFLICT OF INTEREST STATEMENT

M Lehmann is an employee of Varian Medical Systems

## References

[1] Li H , Giles W , Ren L , Bowsher J , Yin FF . Implementation of dual‐energy technique for virtual monochromatic and linearly mixed CBCTs. Med Phys. 2012;39(10):6056‐6064. doi:10.1118/1.4752212 23039644

[2] Sajja S , Lee Y , Eriksson M , et al. Technical principles of dual‐energy cone beam computed tomography and clinical applications for radiation therapy. Adv Radiat Oncol. 2020;5(1):1‐16. doi:10.1016/j.adro.2019.07.013 32051885 PMC7004939

[3] Keeler A , Lehmann M , Luce J , Kaur M , Roeske J , Kang H . Technical note: TIGRE‐DE for the creation of virtual monoenergetic images from dual‐energy cone‐beam CT. Med Phys. 2024;51(4):2975‐2982. doi:10.1002/mp.17002 38408013 PMC10994758

[4] Cassetta R , Lehmann M , Haytmyradov M , et al. Fast‐switching dual energy cone beam computed tomography using the on‐board imager of a commercial linear accelerator. Phys Med Biol. 2020;65(1):015013. doi:10.1088/1361-6560/ab5c35 31775131 PMC7043019

[5] Keeler A , Luce J , Lehmann M , Roeske JC , Kang H . Fast, automated optimization of virtual monoenergetic images with the dual‐energy image synthesizer for cone‐beam CT. J Appl Clin Med Phys. Published online April 22, 2025. doi:10.1002/acm2.70083 PMC12148776 40260755

[6] Gang GJ , Zbijewski W , Webster Stayman J , Siewerdsen JH . Cascaded systems analysis of noise and detectability in dual‐energy cone‐beam CT. Med Phys. 2012;39(8):5145‐5156. doi:10.1118/1.4736420 22894440 PMC3422357

[7] Iramina H , Hamaguchi T , Nakamura M , Mizowaki T , Kanno I . Metal artifact reduction by filter‐based dual‐energy cone‐beam computed tomography on a bench‐top micro‐CBCT system: concept and demonstration. J Radiat Res. 2018;59(4):511‐520. doi:10.1093/jrr/rry034 29718315 PMC6054224

[8] Deng Y , Zhou H , Wang Z , Wang AS , Gao H . Multi‐energy blended CBCT spectral imaging and scatter‐decoupled material decomposition using a spectral modulator with flying focal spot (SMFFS). Med Phys. 2024;51(4):2398‐2412. doi:10.1002/mp.17022 38477717

[9] Ozoemelam I , Myronakis M , Harris TC , et al. Monte Carlo model of a prototype flat‐panel detector for multi‐energy applications in radiotherapy. Med Phys. Published online October 1, 2023. doi:10.1002/mp.16689 37665764

[10] Wang Z , Zhou H , Gu S , et al. Dual‐energy head cone‐beam CT using a dual‐layer flat‐panel detector: hybrid material decomposition and a feasibility study. Med Phys. 2023;50(11):6762‐6778. doi:10.1002/mp.16711 37675888

[11] Altunbas C . Feasibility of dual‐energy CBCT material decomposition in the human torso with 2D anti‐scatter grids and grid‐based scatter sampling. Med Phys. 2024;51(1):334‐347. doi:10.1002/mp.16611 37477550 PMC11009009

[12] Skaarup M , Edmund JM , Dorn S , Kachelriess M , Vogelius IR . Dual‐energy material decomposition for cone‐beam computed tomography in image‐guided radiotherapy. Acta Oncol (Madr). 2019;58(10):1483‐1488. doi:10.1080/0284186X.2019.1629010 31271086

[13] Li M , Zhao Y , Zhang P . Accurate iterative FBP reconstruction method for material decomposition of dual energy CT. IEEE Trans Med Imaging. 2019;38(3):802‐812. doi:10.1109/TMI.2018.2872885 30281441

[14] Dong X , Niu T , Zhu L . Combined iterative reconstruction and image‐domain decomposition for dual energy CT using total‐variation regularization. Med Phys. 2014;41(5):051909. doi:10.1118/1.4870375 24784388

[15] Li C , Zhou L , Deng J , et al. A generalizable new figure of merit for dose optimization in dual energy cone beam CT scanning protocols. Phys Med Biol. 2023;68(18):185021. doi:10.1088/1361-6560/acf3cd 37619587

[16] Zbijewski W , Gang GJ , Xu J , et al. Dual‐energy cone‐beam CT with a flat‐panel detector: effect of reconstruction algorithm on material classification. Med Phys. 2014;41(2):021908.doi:10.1118/1.4863598 24506629 PMC3977791

[17] Ren L , Rajendran K , McCollough CH , Yu L . Quantitative accuracy and dose efficiency of dual‐contrast imaging using dual‐energy CT: a phantom study. Med Phys. 2020;47(2):441‐456. doi:10.1002/mp.13912 31705664 PMC7015798

[18] Vilches‐Freixas G , Létang JM , Ducros N , Rit S . Optimization of dual‐energy CT acquisitions for proton therapy using projection‐based decomposition: Med Phys. 2017;44(9):4548‐4558. doi:10.1002/mp.12448 28675582

[19] Shkumat NA , Siewerdsen JH , Dhanantwari AC , et al. Optimization of image acquisition techniques for dual‐energy imaging of the chest. Med Phys. 2007;34(10):3904‐3915. doi:10.1118/1.2777278 17985636

[20] Gros S , Bian J , Jackson J , et al. Proposal and evaluation of a practical CBCT dose optimization method. arXiv. Preprint posted online February 3, 2025. doi:10.48550/arXiv.2502.01509

[21] Biguri A , Dosanjh M , Hancock S , Soleimani M . TIGRE: A MATLAB‐GPU toolbox for CBCT image reconstruction. Biomed Phys Eng Express. 2016;2(5):055010. doi:10.1088/2057-1976/2/5/055010

[22] Du Y , Wang R , Biguri A , Zhao X , Peng Y , Wu H . TIGRE‐VarianCBCT for on‐board cone‐beam computed tomography, an open‐source toolkit for imaging, dosimetry and clinical research. Phys Med. 2022;102:33‐45. doi:10.1016/j.ejmp.2022.08.013 36088800

